# Dietary Fibre as a Unifying Remedy for the Whole Spectrum of Obesity-Associated Cardiovascular Risk

**DOI:** 10.3390/nu10070943

**Published:** 2018-07-21

**Authors:** Lutgarda Bozzetto, Giuseppina Costabile, Giuseppe Della Pepa, Paola Ciciola, Claudia Vetrani, Marilena Vitale, Angela A. Rivellese, Giovanni Annuzzi

**Affiliations:** Department of Clinical Medicine and Surgery, Federico II University, 80131 Naples, Italy; giuseppina.costabile@unina.it (G.C.); giuseppe.dellapepa@unina.it (G.D.P.); paola.ciciola@gmail.com (P.C.); c.vetrani@libero.it (C.V.); marilena.vitale@yahoo.it (M.V.); rivelles@unina.it (A.A.R.); annuzzi@unina.it (G.A.)

**Keywords:** dietary fibre, obesity, diabetes type 2, hypertension, dyslipidemia, cardiovascular risk, metabolic syndrome, insulin resistance

## Abstract

Obesity is a pandemic carrying the heavy burden of multiple and serious co-morbidities including metabolic syndrome, type 2 diabetes and cardiovascular diseases. The pathophysiological processes leading to the accumulation of body fat slowly evolve to fat accumulation in other body compartments than subcutaneous tissue. This abnormal fat deposition determines insulin resistance which in turn causes blood glucose and lipid metabolism derangement, non-alcoholic fatty liver disease, hypertension, and metabolic syndrome. All these conditions contribute to increase the cardiovascular risk of obese people. Several randomized clinical trials demonstrated that moderate weight loss (5–10%) in obese patients improves obesity-related metabolic risk factors and coexisting disorders. Therefore, nutritional strategies able to facilitate weight management, and in the meantime positively influence obesity-associated cardiovascular risk factors, should be implemented. To this aim, a suitable option could be dietary fibres that may also act independently of weight loss. The present narrative review summarizes the current evidence about the effects of dietary fibres on weight management in obese people. Moreover, all of the different cardiovascular risk factors are individually considered and evidence on cardiovascular outcomes is summarized. We also describe the plausible mechanisms by which different dietary fibres could modulate cardio-metabolic risk factors. Overall, despite both epidemiological and intervention studies on weight loss that show statistically significant but negligible clinical effects, dietary fibres seem to have a beneficial impact on main pathophysiological pathways involved in cardiovascular risk (i.e., insulin resistance, renin-angiotensin, and sympathetic nervous systems). Although the evidence is not conclusive, this suggests that fibre would be a suitable option to counteract obesity-related cardio-metabolic diseases also independently of weight loss. However, evidence is not consistent for the different risk factors, with clear beneficial effects shown on blood glucose metabolism and Low Density Lipoprotein (LDL) cholesterol while there is fewer, and less consistent data shown on plasma triglyceride and blood pressure. Ascribing the beneficial effect of some foods (i.e., fruits and vegetables) solely to their fibre content requires more investigation on the pathophysiological role of other dietary components, such as polyphenols.

## 1. Introduction

### 1.1. Pathophysiology of Obesity and Related Cardiovascular Risk Factors

Obesity is a worldwide pandemic currently involving 600 million adults and 40 million children prior to 5 years of age [1]. The obesity-related burden is mainly carried out by multiple, serious co-morbidities including metabolic syndrome, type 2 diabetes and cardiovascular diseases.

The pathophysiological processes leading to the aberrant accumulation of body fat evolve slowly over time driven by the interaction of gene and environment in the complex system that regulates energy balance [2,3]. Both central and peripheral signals are involved in the regulation of short and long–term energy balance. Central signals start from brain regions inside and outside the hypothalamus presiding over cognitive processes, hedonic effects of food consumption, memory, and attention. Peripheral signals arise from each district of the body including the microbiome and cells within adipose tissue, stomach, pancreas, and other organs [3].

The disruption of this intricate network that physiologically counteracts variations of food intake and/or physical activity in order to keep body weight constant leads to the constitutional activation of orexigenic signals with a chronic positive energy balance leading to the deposition of energy oversupply in triglyceride form.

Adipose tissue is physiologically deputed to store lipids at a variety of anatomical sites that differ in metabolic and physiological characteristics. Subcutaneous adipose tissue is the body compartment with the highest fat storing capacity. Obesity occurs when fat amount exceeds the holding ability of subcutaneous depots. This determines the inflammation of adipose tissue, which contributes to the insulin resistance that is often present in patients with obesity [3,4].

The threshold saturation of subcutaneous adipose tissue is mainly genetically determined and is associated with advanced disruption of glucose metabolism such as type 2 diabetes [5].

**Figure 1:**
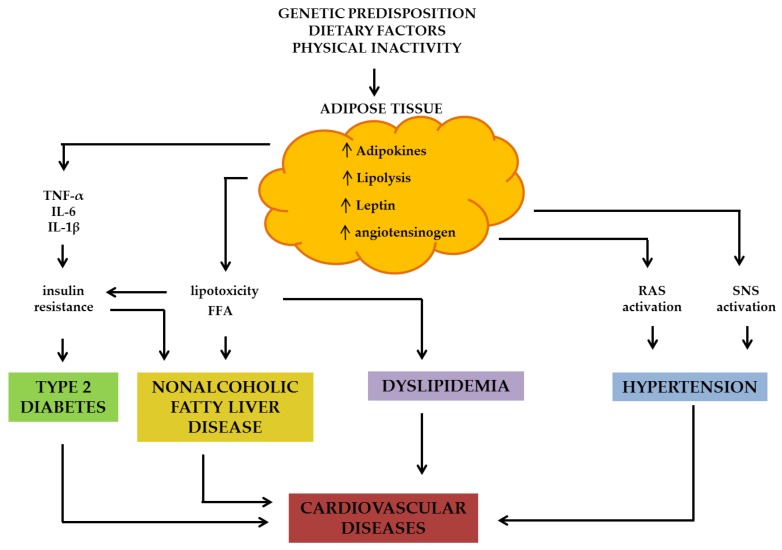
Pathophysiological mechanisms by which excessive adipose tissue leads to metabolic dysfunction and common chronic diseases. RAS: renin-angiotensin system; SNS: sympathetic nervous system; FFA: free fatty acids; IL-6: interleukin-6; IL-1β: interleukin-1β; TNF-α: tumor necrosis factor-α.

Visceral adipose tissue is a less wide compartment whose expansion, in particular at mesenchymal and omental level, is associated with higher risk of diabetes and cardiovascular disease. The “overcrowded” subcutaneous adipose tissue and the visceral fat are dysfunctional compartments with abnormal enzymatic lipolytic activities [6], secreting inappropriate types and amounts of cytokines and adipokines that regulate glucose and lipids metabolism with paracrine and endocrine mechanisms (Figure 1).

The first consequence of adipose tissue dysfunction is the inappropriate lipolysis of stored triglycerides. The resultant increase of circulating free fatty acids, in turn, determines insulin-resistance, deregulation of hepatic glucose metabolism and eventually, the occurrence of diabetes [7,8]. The higher concentration of free fatty acids, together with the stimulation of *de novo lipogenesis* and the inhibition of beta-oxidation and hepatic Very Low Density Lipoprotein (VLDL) secretion, makes available more substrate for triglyceride synthesis in the liver. The hepatic deposition of the triglyceride surplus determines non-alcoholic fatty liver disease, an ominous condition potentially evolving in end-stage liver disease and associated with a higher risk of diabetes and cardiovascular disease, characterized by a wide spectrum of hepatic histologic abnormalities ranging from simple steatosis to cirrhosis [9].

Both adipose tissue dysfunction and ectopic fat deposition contribute to the genesis of atherosclerotic dyslipidemia, i.e., high plasma triglyceride; low High Density Lipoprotein (HDL) cholesterol; high LDL cholesterol; fasting; and postprandial alterations of lipoprotein composition associated with cardiovascular disease [10,11,12].

Obesity is often associated with chronic over-activity of the sympathetic nervous system. This, in combination with the activation of renin–angiotensin–aldosterone system and mechanical stress-inducing renal compression, may account for the high prevalence of high blood pressure in obese people [13].

### 1.2. Dietary Fibre

Dietary fibre is defined as “the edible part of plants or their extracts, or analogous carbohydrates, that are resistant to digestion and absorption in the human small intestine, and undergoes complete or partial fermentation in the large intestine” [14]. Several classification systems have been proposed for dietary fibres. Taking into account their main properties (i.e., viscosity, water solubility, and fermentation rate), dietary fibre can be classified into four main groups, as reported in Table 1, in which we summarized the concepts of McRorie et al. [15].

1. Non-viscous, insoluble, non-fermentable fibre. It is an insoluble fibre that is very poorly fermented in the intestine. It has mechanical and laxative effects, contributing to regulate digestive function. Bran, cellulose, hemicellulose and lignin are the main fibres representative of this group.
2. Non-viscous, soluble, fermentable fibre. It is quickly and greatly fermented by the intestinal microbiota. It may have a prebiotic effect, but it does not induce laxative effects. Inulin, dextrin, oligosaccharides, resistant starch are the main fibres representative of this group.
3. Viscous, soluble, fermentable fibre. It can form a viscous gel that reduces nutrient absorption in the intestine. Moreover, it is rapidly fermented by the intestinal microbiota. Pectin, β-glucan, guar gum, and glucomannan are the main fibres representative of this group.
4. Viscous, soluble, non-fermentable fibre. It reduces the absorption of nutrients due to its viscosity and exerts laxative effects. *Psyllium* and methylcellulose are the main fibres representative of this group.

Several randomized clinical trials demonstrated that moderate weight loss (5–10%) in obese patients improves obesity-related metabolic risk factors and coexisting disorders [16,17,18]. Therefore, nutritional strategies able to modify, induce and maintain weight loss should be implemented. These strategies may need qualitative dietary modifications that could favorably affect mechanisms behind obesity and associated cardiometabolic risk factors development. Increasing the consumption of dietary fibre may be a strategy contributing to this purpose.

Thus, the aim of the present narrative review is to summarize the current evidence on the effects of dietary fibres on (a) weight management in obese people and (b) obesity-associated cardio-metabolic diseases. We have comprehensively examined the evidence from observational studies and randomized controlled trials performed in humans to evaluate the effects of fibres on clinical outcomes including cardiovascular events. We also describe the plausible mechanisms by which dietary fibres could modulate cardio-metabolic outcomes.

Literature searching for this narrative review was conducted by searching PubMed databases for epidemiological studies, randomized controlled clinical trials, and meta-analyses on adults published in the English language, during the last 10 years, also considering, if available, previous most relevant reference trials. We used as keywords “detary fibre OR fibre” and separate search terms for obesity and each of the cardiovascular risk factors. The search yielded 531 articles for “obesity OR weight management OR waist circumference OR body weight OR abdominal obesity”, 397 articles for “insulin resistance OR blood glucose OR diabetes risk”, 577 articles for “dyslipidemia OR lipids OR cholesterol OR triglycerides”, 132 articles for “cardiovascular risk OR cardiovascular risk factors”, 32 studies for “non alcoholic fatty liver disease”. This narrative review includes: 1) the meta-analyses of randomized clinical trials; 2) clinical trials not available in the meta-analyses that added significant information and are published in journals in the highest impact factor quartile in the “Endocrinology and Metabolism” or “Nutrition and Dietetics“ areas; 3) epidemiological studies published in journals in the highest impact factor quartile in the “Endocrinology and Metabolism” or “Nutrition and Dietetics” areas. We excluded studies not specifically related to each issue of interest and reviews.

## 2. Body Weight Regulation

### 2.1. Epidemiological Studies

Data from nine large epidemiological studies, two cross-sectional [19,20] and seven longitudinal studies [21,22,23,24,25,26,27] consistently demonstrate that fibre intake is favourably associated with outcomes related to weight management (i.e., BMI, body weight, percentage of body fat, and waist circumference). Longitudinal studies clearly demonstrate that high consumption of dietary fibre, generally more than 10 g/1000 kcal/day or about 20 g/day in most studies, is associated with a body weight loss ranging from 1.2 to 3.6 kg in 8–12 years of follow-up [21,22,23]. In the EPIC-Potsdam cohort, beneficial effects of fibres on body weight also persisted over time as shown by the fact that individuals with the greatest intake of dietary fibre (13.5 g/1000 kcal/day) were more able to maintain body weight or to prevent excess weight gain over 4 years of follow-up than to those with the smallest intake (8.8 g/1.000 kcal/day) [24]. Beneficial effects of fibres on body weight were observed in all cohorts irrespective of baseline body weight status (normal-weight, overweight or obese) of the study participants.

Observational studies also show that a higher intake of dietary fibres is associated with a healthier distribution of body fat. In a cross-sectional observation [19], individuals in the highest quintile of fibre intake (11.3 g/1000 kcal) compared with those in the lowest quintile (6.6 g/1000 kcal) had a smaller waist circumference (82.9 vs. 86.1 cm). In a longitudinal study, overweight youth who had decreased dietary fibre intake from year 1 to year 2 (mean decrease 3 g/1000 kcal) compared with participants who had increased dietary fibre intake (mean increase 3 g/1000 kcal) had a significant increase in visceral adipose tissue (+21% vs. −4%) [25]. Similarly, an inverse association between cereal fibre intake (highest quintile *vs.* lowest quintile) with percent body fat (34.7% (32.8–36.6) vs. 31.5% (29.4–33.5); *P*_trend_ = 0.004) and trunk fat mass (42.8% (40.2–45.4) vs. 37.8% (35.0–40.6); *P*_trend_ = 0.001) was shown by McKeown et al. [20].

Epidemiological evidence also shows a beneficial effect of fibre on weight management considering total dietary fibre or cereal fibre intake. In a 6.5-year follow-up large European study, 10 g/day of total fibre intake was associated with a weight reduction of 39 g/year and a waist circumference reduction of 0.08 cm/year, while 10 g/day in whole grain fibre intake was associated with a weight reduction of 77 g/year and a waist circumference reduction of 0.10 cm/year [26]. In The Health Professionals Follow-up Study, over an 8-year follow-up period, for each 20 g/day increase in total dietary fibres, weight gain was reduced by 1.2 kg, while for each 20 g/day increase in cereal fibres weight gain was reduced by 0.81 kg [27].

In all the epidemiological studies reported above, the adjustment for confounders, including other dietary components and energy intake, did not influence or only modestly attenuated the association between dietary fibre intake and weight outcomes.

### 2.2. Randomized Controlled Trials

In the last decades, a large amount of clinical trials have been conducted to investigate the possible effects of fibre on outcomes related to body weight changes. These trials focused on the effects of different types of fibre—i.e., fibres from whole grain, pulses, fruit and vegetables, and fibre supplements—and some of these trials have been included in previously published meta-analyses reported in Table 2.

In contrast with epidemiological evidence, the results of clinical trials evaluating the effect of fibres from whole grain on weight reduction are limited and not consistent. In a meta-analysis of 26 studies, Pol et al. [28] showed that whole grain intake compared to refined grain had no effect on body weight, although it slightly reduced the percentage of body fat. Seven of these trials compared whole with refined grain in weight-loss diets, while the other 19 studies were performed on isoenergetic conditions. The daily whole grain intake ranged from 18.2 to 150 g/day and the trials’ duration ranged from 2 to 16 weeks. In a “review of meta-analyses” [32], the reductions in body weight, BMI, and abdominal obesity observed in individuals with the highest dietary whole grain intake were not significantly different from individuals consuming non-whole grain foods or refined grain. In a more recent RCT, not included in the previous meta-analysis, Kirwan et al. [33] showed that an 8-week whole grain vs. refined grain diet (29 vs. 21 g/day fibre intake) in 40 overweight/obese adults induced a similar effect on weight loss (−3%), fat loss (−6%), and waist circumference (−2 cm) with no significant difference between diets. The discrepancy between epidemiological studies and RCTs could be related to differences in study design, short duration of trials, selected populations, and the type and amount of whole grain foods consumed.

With regard to fibre from pulses, a meta-analysis of 21 trials showed a significant weight reduction of 0.34 kg in diets containing dietary pulses (median intake of 132 g/day or about 1 serving/day) compared with diets without dietary pulse, over a median duration of 6 weeks [29]. The effect on body weight reduction was observed in trials testing both low-calories and no calorie restricted diets; in 6 trials, a reduction in body fat percentage (−0.34%) was also observed [29].

With respect to fruit and vegetables, the meta-analysis by Mytton et al. [30], including 8 randomized trials, showed a significant weight reduction of 0.68 kg with diets rich in fruit and vegetables (in the absence of specific advice to decrease consumption of other foods) compared to diets poor in fruit and vegetables. The study duration ranged from 4 to 52 weeks and the mean difference in high vs. low fruit and vegetables intake for trial’s arms was 133 g/day (ranging from 50 to 456 g).

Concerning the effects on body weight of fibre from pulses, and even more from fruits and vegetables, the influence of other components such as polyphenols should be considered.

Studies on the effects of soluble fibre supplementation seem to support a *per se* beneficial effect of this type of fibre. Thompson et al. [31] in a meta-analysis of 12 RCTs in overweight and obese adults, reported that soluble fibre supplementation, compared to placebo, reduced BMI by 0.84 kg/m^2^, body weight by 2.52 kg, and body fat by 0.41%. The soluble fibres were used as supplements in the form of manno-oligosaccharides, galacto-oligosaccharides, fructo-oligosaccharides, β-glucan, flaxseed mucilage, mannans and dextrin. The mean soluble fibre dose was 18.5 g/day (range: 3–34 g/day) and the trials’ duration ranged from 2 to 17 weeks (Table 2).

In conclusion, evidence from RCTs indicates that low-fermentable fibres intake, mostly from whole grain, does not affect body weight; this could be related to the heterogeneity of experimental settings in which whole grains have been tested. Fermentable fibres from other sources (pulses, fruits and vegetables) induced a statistically significant but clinically irrelevant reduction in body weight that, however, was prevalently obtained in isocaloric conditions.

### 2.3. Possible Mechanisms of Fibre Effects on Body Weight Regulation

In the small intestine, by creating a mechanical barrier and increasing intraluminal viscosity, soluble and insoluble fibre delay intestinal transit and reduce glucose and free fatty acid absorption with a consequent increment in fat oxidation and reduction in fat storage. Furthermore, the reduction in glucose absorption can also decrease insulin secretion, preventing the risk of reactive hypoglycemia during the post-absorption period and reducing hunger [34,35]. In the small intestine, the role of dietary fibre on gastrointestinal hormone secretion should be also considered. In particular, a fibre-rich meal may favour the release of cholecystokinin, a peptide involved in gastric emptying regulation and hypothalamic satiety nucleus stimulation [36].

Dietary fibre also increases glucagon-like peptide-1, a gut hormone involved in satiety control, gastric emptying and small intestine transit [37]. Finally, in the large intestine, fibre is fermented by intestinal bacteria and influences microbiota composition [38,39]. Short-chain fatty acids (SCFAs) derived from intestinal bacteria (acetic, propionic and butyric acids) could positively influence body weight regulation by different mechanisms: decreasing gastric emptying and prolonging satiety, improving insulin sensitivity and modulating glucose and lipid oxidation [40,41,42]. With respect to microbiota composition, dietary fibre can increase *Bacteroidetes* and *Actinobacteria*, which are predominant in lean individuals, and decrease the prevalence of *Firmicutes* and *Proteobacteria*, which are dominant in obese individuals [43]; the beneficial impact on microbiota could explain the possible fibre effect on body weight regulation mediated by increasing caloric extraction from food [44].

## 3. Insulin Resistance, Type 2 Diabetes Risk, and Blood Glucose Control in Diabetes

### 3.1. Epidemiological Studies

To the best of our knowledge, epidemiological evidence on the association between dietary fibre intake and insulin resistance only comes from a secondary analysis of the Insulin Resistance Atherosclerosis Study [45]. The results of this US cross-sectional study showed that higher intakes of whole grains were associated with increased insulin sensitivity, evaluated by minimal model analyses of the frequently sampled intravenous-glucose-tolerance test. This association remained statistically significant also after adjusting for potential confounders.

### 3.2. Randomized Controlled Trials

The available evidence from clinical trials on insulin resistance comes mainly from studies investigating the effects of whole grains (Table 3). Since, to the best of our knowledge, no meta-analysis on the effect of fibre from whole grain specifically on insulin-resistance exists, we reported the evidence by individual RCTs. With the only exception of the study by Pereira and colleagues [46] who observed a significant reduction of insulin resistance after a 6-week diet based on wholegrain products, trials evaluating the effect of wholegrain have consistently found no changes of insulin resistance/sensitivity in overweight/obese people [47,48,49,50,51,52,53,54]. This lack of effects on insulin resistance from wholegrains was observed independently of (1) the methodology applied for insulin resistance measurement, (2) body weight changes after the intervention, and (3) exposure time to fibre consumption (ranging from 3 to 16 weeks).

Looking at other types of fibre, the main findings allow different conclusions to be drawn. In a meta-analysis evaluating the effects of cereal fibre (β-glucan) on HOMA index, He et al. [55] concluded that oat-based products, β-glucan extract, and refined-grain products did not differently influence insulin resistance in overweight, otherwise healthy, individuals. These results are in contrast with two previous trials evaluating the effects of a 12-week supplementation of soluble fibres (resistant maltodextrin [56] and NUTRIOSE [57]) that detected a significant reduction of insulin resistance evaluated by the HOMA index. Finally, as observed in two studies [58,59], resistant starch supplementation (40 g/day) seems to be effective in improving insulin resistance/sensitivity. The effects of soluble fibre and resistant starch on insulin-resistance were observed independently of body weight changes as reported in Table 3.

### 3.3. Possible Mechanisms of Fibre Effects on Insulin Resistance

Mechanisms underlying the effects of soluble fibre consumption on insulin resistance can be firstly ascribed to the fermentation process with the consequent production of SCFAs. Indeed, increased SCFAs production has been related to the improvement of insulin sensitivity in several intervention trials [60,61,62]. Moreover, recent data in animals show that different types of fibre are able to differently influence SCFAs production with consequent different effects on pathophysiological properties of adipose tissue related to insulin resistance such as browning. This would confirm also that independently of the induction of clinically relevant body weight changes, dietary fibre could act on mechanisms behind the association between adiposity and insulin resistance [63].

However, the prebiotic effect of fibre may also play a role. It has been observed that obesity-related dysbiosis may lead to the development of insulin resistance [64,65]. Several studies have demonstrated that fibre intake can influence microbiota composition, favouring bacterial species that do not contain the detrimental compounds that trigger endotoxemia (i.e., lipopolysaccharides and peptidoglycans) [66]. Therefore, by relieving endotoxemia, fibre consumption could reduce insulin resistance. It must also be considered that high fibre diets are often lower in fat, with some fats, i.e., saturated and *trans* fatty acids, also known to favour endotoxemia and inflammation leading to insulin resistance.

Finally, animal studies support the activity of fibre in modulating the expression of the insulin-responsive glucose transporter type 4 (GLUT-4) in the skeletal muscle, thus improving insulin sensitivity [67,68].

### 3.4. Diabetes Risk

The beneficial effects of dietary fibre on insulin-resistance imply that they can also impact on diabetes risk. We selected five epidemiological studies suggesting that a high fibre intake reduces the risk of diabetes. One of the first large-scale studies [69] on 65,173 women between 40 and 65 years of age, showed an inverse association between risk of diabetes and fibre intake, mainly from cereals. More recently, a meta-analysis of 19 cohort studies by the InterAct Consortium has shown the consistency of results over time, with a summary diabetes RR per 10 g/day increase in intake of 0.91 (95% CI 0.87, 0.96) for total fibre, 0.75 (95% CI 0.65, 0.86) for cereal fibre, 0.95 (95% CI 0.87, 1.03) for fruit fibre and 0.93 (95% CI 0.82, 1.05) for vegetable fibre [70]. Taken as a whole, these data from cohort studies indicate that insoluble/non-viscous/cereal fibre is protective against diabetes while soluble/viscous/fruit fibre give little protection [69,70]. It is of interest to underline that most of these findings come from cohort studies conducted in the United States (~85%) where cereals are the predominant source of dietary fibre followed by more meagre proportions from vegetables and fruit sources [71,72,73]. In all these epidemiological studies, the adjustment for confounders did not influence or only modestly attenuated the association between dietary fibre intake and diabetes risk.

On the other hand, it should be taken into account that current observational evidence [74,75,76] and the results of a large randomized controlled trial, the PREDIMED study [77], demonstrated an inverse association between the adherence to the Mediterranean diet and diabetes incidence during a median follow-up of 4 years; indicating that this dietary pattern is the most useful dietary approach to reduce diabetes risk. Although it is not possible to isolate the effects of fibre from those of other beneficial components, the high intake of wholegrain cereals, fruits, and vegetables characterising the Mediterranean diet supports a relevant role for all types of dietary fibre in reducing diabetes risk.

### 3.5. Blood Glucose Control

Dietary fibre does not only reduce diabetes risk but also influences blood glucose control in diabetes. Our search revealed 215 studies on this topic, but only one cross-sectional and six meta-analyses of RCTs were included. Vitale and colleagues [78] showed in a cross-sectional analysis on 2573 people with type 2 diabetes that a consumption of fibre ≥15 g/1000 kcal was associated with a significantly lower HbA1c compared with a fibre intake <10 g/1000 kcal. In line with these findings, two meta-analysis of RCT studies in people with type 2 diabetes have shown that increasing fibre intake, in particular soluble fibres, significantly decreases blood glucose concentration and HbA1c levels [79,80] (Table 4).

Looking at specific type of fibres, *psyllium* supplementation has shown to significantly improve fasting glucose and HbA1c concentrations in type 2 diabetic patients [81]. Fruit consumption has also been inversely associated with blood glucose control, which may be mediated in part by dietary fibre, although studies in this area are somewhat lacking [84,85].

Similarly to the effects on diabetes risk, the Mediterranean diet, with its high fibre content together with other beneficial components, has been shown to reduce fasting plasma glucose and Hb1Ac in a recent meta-analysis by Schwingshackl et al. [82] on 4937 men and women with type 2 diabetes.

Direct effects of dietary fibre on postprandial glucose metabolism are also supported by the only study available on patients with type 1 diabetes in which a high-fibre (20.8 g) meal acutely blunted postprandial blood glucose response and reduced pre-meal insulin needs with respect to a low-fibre meal (7.8 g) [86].

As for healthy individuals, the meta-analysis of Marventano et al. [83], assessing the acute and medium/long-term effects on blood glucose levels in 1033 subjects, showed significant reductions of the glucose post-prandial iAUC (0–120 min) by −29.7 mmol min/L (95% CI: −43.56, −15.8), and the insulin post-prandial iAUC (0–120 min) by −2.01 nmol min/L (95% CI: −2.88, −1.14) with wholegrain foods in acute tests but no effects on fasting glucose and insulin in medium-term interventions.

A recent meta-analysis [31] focusing on blood glucose metabolism effects of soluble fibre supplementation showed that fibre supplements, independently of caloric restriction, improved insulin resistance and reduced fasting blood glucose and insulin concentrations by 0.17 mmol/L and 15.9 pmol/L, respectively, in overweight/obese people. Moreover, fibre-type-based meta-regressions showed a greater effect for non-viscous fermentable soluble fibres than viscous fermentable fibres [31].

### 3.6. Possible Mechanisms of Fibre Effects on Blood Glucose Control

The potential mechanisms of action of dietary fibre on glycaemic control include delay of gastric emptying; effect on hormonal regulation of digestion and absorption; alteration of enzymatic action, particularly amylases; and delay of sugar absorption through reduction in rates of diffusion, interactions with the mucosa or down-regulation of glucose transporters. These are linked to the physical-chemical properties of dietary fibres, particularly the ability of some dietary fibres to increase viscosity of intestinal contents, thus offering numerous opportunities to affect the regulation of glucose and insulin metabolism [18].

### 3.7. Conclusions

Epidemiological and intervention trials data are not consistent for all type of dietary fibres when showing beneficial effects on insulin resistance, risk of diabetes and glucose metabolism in healthy and mainly in obese individuals with or without diabetes. The epidemiological data suggest that low-fermentable cereal fibre is more suitable for reducing the risk of diabetes as well as for glycaemic control than other sources. RCTs show conflicting results (Table 3), most of them showing that foods containing viscous fibre may reduce the glycaemic response whereas foods that are rich in insoluble/non viscous, low-fermentable fibre, such as whole wheat, have little effect on blood glucose levels. Therefore, taking into account the beneficial effects of fibre intake on insulin resistance—i.e., the main pathophysiological process leading to blood glucose disruption in obesity (Figure 1), and on blood glucose control, the consumption of foods rich in fibre should be encouraged to prevent or improve blood glucose metabolism derangement that typically occurs in people with obesity.

## 4. Dyslipidaemia

### 4.1. Epidemiological Studies

Two epidemiological studies met the study’s inclusion criteria. A cross-sectional analysis in 22,915 participants of the EPIC Norfolk study showed that a mean fibre intake of 18.5 g/day was inversely associated with serum cholesterol and triglycerides levels [87]. Recently, Vitale et al. [88] in type 2 diabetes patients enrolled from the TOSCA.IT study, observed that in a sub-group of subjects not on any lipid-lowering drug, dietary fibre intake was significantly associated with lower LDL-cholesterol and triglyceride plasma levels. These associations remained statistically significant also after the adjustment for multiple confounders.

### 4.2. Randomized Controlled Trials

In recent years, several intervention trials focused on the cholesterol-lowering effects of diets rich in different types of fibre (Table 5). Overall, the body of evidence from RCTs shows that fibre-enriched diets, mainly characterized by higher intake of legumes, fruit and vegetables, reduces LDL-cholesterol plasma levels.

Data on the effects of wholegrain-based diets on plasma lipid levels are still limited and controversial (Table 5). Indeed, a cholesterol-lowering effect of wholegrain intake has been found in some studies [89,90]; however, this reduction did not reach the statistical significance in all meta-analyses of RCTs [91,92]. The lack of conclusive evidence may be due to the huge variability of grain types, doses and time of exposure in the different studies.

Among grain-derived fibres, only β-glucans from oat and barley, i.e., soluble fibre, have been demonstrated to improve lipids concentrations. In particular, a β-glucans dose > 3 g/day was indicated as the effective dose to achieve a significant reduction of LDL-cholesterol concentration [93,94,95,96].

In a meta-analysis of intervention studies with legume-rich diets (80–440 g/day), characterized by high amount of psyllium, pectin and guar gum, a significant reduction of LDL-cholesterol by −8.0 mg/dL was observed [97].

The cholesterol-lowering effects have also been ascribed to supplements of other soluble fibres from fruits, vegetables and legumes, such as pectin, guar gum, and psyllium [98,99]. The cholesterol-lowering effects of these fibres given as supplements have been summarized by Pirro et al. [100], who reported that soluble fibre supplementation reduced plasma LDL-cholesterol levels on average by 4% to 14%, with possible variations related to the different doses used in the various studies.

Finally, a recent meta-analysis of 20 RCTs has shown that inulin-type fructans (mean dose: 14 g/day) may significantly reduce LDL-cholesterol concentrations [101], with no effect on the other lipids (HDL-cholesterol and triglycerides).

As for the effect of fibre-rich foods or fibre supplementation on HDL-cholesterol, the overall evidence indicates no relevant effects (Table 5).

As for plasma triglyceride levels, while no effect has been detected on fasting triglycerides (Table 5), several studies have demonstrated a triglyceride-lowering effect of fibre during the postprandial period. In particular, a 4-week fibre-rich diet (mainly soluble fibre, 28 g/1000 kcal) significantly affected chylomicron-triglyceride response in overweight-obese patients with type 2 diabetes [102,103,104]. A relevant reduction of postprandial plasma triglyceride response was also observed in subjects with metabolic syndrome after a 12-week wholegrain rich-diet [54]. However, further studies are needed to clarify this issue.

### 4.3. Possible Mechanisms of Fibre Effects on Dyslipidaemia.

The mechanisms that have been proposed to explain the effect of soluble fibre on LDL cholesterol levels may be linked to its viscosity that inhibits the intestinal uptake of dietary cholesterol and the reabsorption of bile [105]. Furthermore, soluble fibre may increase faecal excretion of bile acids and enhance hepatic conversion of cholesterol into bile acids. Finally, soluble fibre may be fermented by colonic microbiota with the production of short-chain fatty acids that have shown to inhibit hepatic cholesterol synthesis [105]. Acting on intestinal cholesterol absorption, fibre intake could not only reduce LDL cholesterol, but also blunt atherogenic dyslipidaemia—high triglycerides, postprandial lipoproteins/remnants—by reducing substrate availability for hepatic lipoprotein production that is generally increased in obesity. This was previously shown in a postprandial study with ezetimibe—an intestinal-cholesterol inhibitor—in overweight patients with type 2 diabetes [106]. Moreover, as previously shown in humans, the reduction of plasma cholesterol absorption by means of a holistic nutritional approach including increased consumption of soluble fibres also reduces the small and dense LDL fraction that is increased in all insulin-resistant states including obesity [107].

As for the effects on postprandial triglycerides, it has been suggested that dietary fibre slows down and reduces the absorption of fat in the small intestine, thus decreasing the production of chylomicrons [108]. Moreover, because of the improved postprandial insulin sensitivity [54, 102] and the consequent reduction of free fatty acid spill-over from adipose tissue and inhibition of *de novo lipogenesis* and ApoB100 synthesis, VLDL production is also reduced. This reduced production of both intestinal and hepatic lipoproteins is a main pathway through which dietary fibres disrupt deleterious mechanisms leading to obesity-related atherogenic dyslipidaemia.

### 4.4. Conclusions

Soluble/high fermentable fibre intake represents an important dietary strategy to influence plasma cholesterol levels in healthy, diabetic, dyslipidemic, and high cardio-metabolic risk individuals. In particular, a well-documented LDL-cholesterol-lowering effect has been demonstrated for added fibre. Very few data are available for other components of atherogenic dislipidemia associated with obesity: high triglyceride and low HDL cholesterol levels.

## 5. Blood Pressure/Hypertension

### 5.1. Epidemiological Studies

Hypertension is a major independent risk factor for CVD [109]. We selected five epidemiological studies highlighting the relationship between dietary fibre consumption and blood pressure levels. Data from the French Nutrition and Health Survey (ENNS 2006–2007), including 18–74 year old participants, showed that dietary fibre and whole grains were inversely and linearly associated with systolic blood pressure [110]. In line with this trend, results from a prospective study in adolescent females showed that a 7.1 g/day increase in dietary intake of total fibre was associated with a 0.96 and 0.62 mmHg decrease in mean systolic and diastolic blood pressures, respectively, 5 years later [111]. Recently, Aljuraiban and colleagues [112] investigated the cross-sectional associations between BP and total, insoluble and soluble fibre intake among 2195 free-living US participants, aged 40–59 years, from the INTERnational study on Macro/micronutrients and blood Pressure (INTERMAP) study. After multivariable adjustment for possible dietary and other lifestyle confounders, the results showed that a higher total fibre intake of 6.8 g/1000 kcal was associated with a 1.69 mmHg lower systolic blood pressure (SBP; 95% CI: −2.97, −0.41) and a higher insoluble fibre intake of 4.6 g/1000 kcal was associated with a 1.81 mmHg lower SBP (95% CI: −3.65, 0.04), whereas soluble fibre was not associated with BP. Raw fruit was the main source of total and insoluble fibre, followed by whole grains and vegetables.

These results are in line with findings of a previous cross-sectional analysis including 5961 middle-aged men and women of the Supplementation in Vitamins and Mineral Antioxidants (SUVIMAX) study, reporting that participants in the highest quartiles had a lower risk of hypertension of 29% for total and 32% for insoluble dietary fibre intakes, with no association reported for soluble fibre intake. In particular, the results showed that 5 g/day higher total fibre intake was associated with a 12% lower risk of hypertension [113].

As for whole-grain, a large prospective study in 28,926 middle-aged and older US health professional women [114], found that a lower risk of hypertension began with whole-grain consumption of 1–2 and 2–4 servings/day or 43–58% of total grains as whole grains. In addition, a whole-grain consumption of >4 servings/day or consumption of 58% of total grains as whole grains, or both, was associated with even greater reductions in the risk of hypertension, suggesting a potential role for increasing whole-grain intake in the primary prevention of hypertension and its cardiovascular complications. The association between hypertension and whole-grain intake remained significant also after an additional adjustment for fibres, suggesting that the protective effect of whole grains may be partly due to the fibrous parts of whole grains. Nevertheless, fibres may not be the only component contributing to a reduced risk of hypertension, because other whole grain components (such as antioxidants, phytosterols, vitamins, and minerals) may also contribute to its beneficial effects on hypertension development. In all the epidemiological studies reported above, the adjustment for confounders did not influence or only modestly attenuated the association between dietary fibre intake and weight outcomes.

### 5.2. Randomized Controlled Trials

According to inclusion criteria, five meta-analyses of RCTs were selected. The evidence from these clinical trials is highly variable rendering the findings inconclusive (Table 6), at least for some types of fibre. Earlier meta-analyses of RCTs showed that total dietary fibre consumption significantly reduced BP, without describing the effects by fibre types [115,116]. Instead, Evans and colleagues [117], performed a detailed analysis of RCTs focusing on the effects of types of fibre differing for their chemical structure on BP in non-diseased populations. The authors found that diets rich in β-glucans, provided as oat bran, oat meals or oat beta-glucan-soluble powder, compared with wheat-based control products, reduced SBP by 2.9 mmHg (95% CI: 0.9–4.9) and DBP by 1.5 mmHg (95% CI: 0.2–2.7) for a median difference in beta-glucans of 4 g, whereas little or no statistical evidence of impact on BP of the other types of dietary fibre was found. A more recently published Cochrane Review showed no effect of soluble fibre on blood pressure [91], suggesting that the effect may depend on the type of viscous soluble fibre. In this regard, a just published systematic review and meta-analysis of RCTs evaluated the BP effects of different types of viscous fibre supplementation (β-glucan from oat and barley, guar gum, *konjac*, pectin and *psyllium*) [118]. Overall, the results showed that viscous fibre reduced SBP (−1.59 mmHg (95% CI: −2.72, −0.46)) and DBP (−0.39 mmHg (95% CI: −0.76, −0.01)) at a median dose of 8.7 g/day (1.45–30 g/day) over a median follow-up of 7 weeks. Within the fibre types, SBP reductions were observed only for supplementation using *psyllium* fibre (−2.39 mmHg (95% CI: −4.62, −0.17)), while for beta-glucan, which also has highly viscous properties, only a trend to SBP and DBP reductions was observed.

The conflicting results may be due to several factors, including the difficulty to isolate the effect of weight reduction from that of fibre supplementation alone; the disparity in clinical blood pressure measuring devices; the high variability of the exposure time to fibre consumption (4–24 weeks); the differences in vehicle of supplementation of dietary fibres (capsules or food sources); and the lack of details about the viscous properties of the consumed fibres.

However, overall findings demonstrate a modest but significant reduction of SBP and DBP following viscous soluble fibre supplementation, in particular beta-glucans from oat and *psyllium*.

### 5.3. Possible Mechanisms of Fibre Effects on Blood Pressure

Several mechanisms may explain the blood pressure lowering effect of soluble viscous fibres including multiple pathways specifically implicated in the insurgence of hypertension in obese people, i.e., sympathetic nervous system and angiotensin system activation (Figure 1). As extensively discussed above, viscous soluble fibres delay gastric emptying and increase satiety and the viscosity of digesta, thus slowing the absorption of glucose and reducing the insulin response in the postprandial phase [114]. Postprandial insulin levels, and more in general, insulin resistance and hyperinsulinemia conditions, may affect blood pressure by increasing renal sodium reabsorption and sympathetic nervous system activation [119,120]. Another mechanism through which viscous fibres, in particular beta-glucans, might improve BP levels may be linked to their LDL-cholesterol-lowering action. Indeed, LDL seems to up-regulate the angiotensin-I receptor gene expression in vascular smooth muscle cells, leading to an elevated functional response of the vascular muscle cell to angiotensin II stimulation, which could conceivably affect BP [121]. Finally, it is also plausible that some other associated beneficial component of viscous soluble fibre-based foods may be responsible for the reduction in BP, such as phenolic compounds.

### 5.4. Conclusions

The overall body of evidence on the effect of fibre intake on blood pressure is not completely conclusive. It suggests a beneficial role of soluble/viscous/high-fermentable fibres on the reduction of SBP and DBP, although definitive conclusions on their specific doses cannot be drawn. However, increasing viscous fibre consumption, when particularly low, may contribute to an additional component in the reduction of CVD risk of obese people by acting, also independently of weight reduction, on specific pathophysiological mechanisms leading to hypertension development in obesity.

## 6. Non Alcoholic Fatty Liver Disease

### 6.1. Epidemiological Studies

Epidemiological studies, in particular one longitudinal, nine case-control and four cross-sectional studies, have shown that fibre intake—from whole grains, fruits and vegetables—in NAFLD patients is lower than in healthy individuals with a mean fibre intake of 22.4 vs. 27.7 g/day, respectively [122,123,124,125]. A review of seven epidemiological studies confirms this trend [126]. Furthermore, an inverse association between fibre-rich dietary patterns—i.e., Mediterranean diet or DASH diet [127]—and NAFLD prevalence has been observed in many epidemiological studies [128]. Although this inverse association between fibre intake and NAFLD prevalence remains statistically significant also after the adjustment for multiple confounders, it is also possible that it may be related to a “multifactorial dietary pattern” in which high fibre content, low glycemic index and low fat content could all together play an important role on the course of NAFLD.

### 6.2. Randomized Controlled trials

Taking into account this consideration, only limited research regarding the effects on NAFLD of fibre alone—i.e., excluding studies in which fibre represented a part of a multifactorial dietary intervention—has been done. To this respect, Bozzetto et al. [129] showed that an 8-week high-fibre low-glycemic index diet did not influence liver fat content in overweight/obese patients with type 2 diabetes. To our knowledge, only one trial investigated the effects of oligofructose, a non-digestible carbohydrate, on NAFLD outcomes [130]. In this 4-week randomized, cross-over trial, a decrease in ALT and AST was observed after 16 g/day of oligofructose intake compared to maltodextrine in patients with NASH. However, no change in liver fat was observed at ultrasound.

In conclusion, epidemiological studies clearly demonstrate that high consumption of dietary fibre, in particular when included in a healthy “multifactorial dietary pattern”, is associated with a lower risk of NAFLD. However, randomized trials sufficiently long, with an adequate sample size and histological endpoints investigating the possible effect of fibre alone on NAFLD, are missing.

### 6.3. Possible Mechanisms of Fibre Effects on NAFLD

Many different mechanisms could explain the potential beneficial role of fibre in NAFLD; most of them—such as lower energy density and satiation leading to body fat reduction—have been described above. Furthermore, fibre can positively affect liver fat content by decreasing glucose absorption, lowering the hepatic influx of glucose and decreasing *de novo lipogenesis* [131]; in addition, the fibre content of foods can positively act on the gut microbiome, a possible mediator by which nutrients may influence liver fat content [132]. Dietary fibres also increase glucagon-like peptide-1 [37], a gut hormone whose decreased postprandial plasma levels were associated with fat content [133].

## 7. Cardiovascular Disease

A systematic review and dose-response meta-analysis of 22 cohort studies, reported an inverse association between total dietary fibre intake and risk of CVD and coronary heart disease (CHD) (RR: 0.91, 95% CI: 0.88–0.94 and 0.91, 95% CI: 0.87–0.94, respectively for each 7 g/day increase in total dietary fibre) [134]. Insoluble fibre and fibre from cereal and vegetable sources were inversely associated with risk of CVD and CHD, whereas fibre from fruit was associated only with a lower CVD risk. In line with this trend, Wu and colleagues [135] focused on the association between different types of fibre (cereal, fruit and vegetable fibre) and the incidence of all coronary events and mortality, quantitatively estimating their dose-response relationships in a meta-analysis of cohort studies. The results showed that especially fibre from cereals and fruits were inversely associated with the incidence of all coronary events (RR: 0.92; 95% CI: 0.85–0.99 and RR: 0.92; 95% CI: 0.86–0.98, respectively) and mortality (RR: 0.81, 95% CI: 0.72–0.92 and RR: 0.68, 95% CI: 0.43–1.07, respectively). Besides, both soluble and insoluble fibres showed a similar and significant inverse association with incidence of CHD and mortality. The favourable inverse association between cereal fibre and CVD risk was also supported by Ye and colleagues [89] in a meta-analysis of prospective cohort studies, in which the authors investigated the relation between whole grain and fibre intake and risk of CVD. The results showed that compared with those who rarely or never consumed whole grains, those reporting an average of 48–80 g/day of whole grains (3–5 serving/day) had a 21% reduction in CVD risk (RR: 0.79, 95% CI: 0.74–0.85). In this study, an inverse association was also found between total and cereal fibre and CVD risk.

Moreover, data from the National Health and Nutrition Examination Survey from 2005 to 2010, in which a total of 11,113 subjects aged 20 to 79 years with no history of CVD, showed that dietary fibre consumption was associated with a low-to-medium overall lifetime CVD risk [136]. This protective effect was extended also to a Mediterranean cohort of elderly adults at high risk for CVD, as reported by an observational cohort analysis of the PREDIMED trial [137] that showed that dietary fibre intake was associated with a reduction in all-cause mortality (RR: 0.63; 95% CI: 0.46–0.86).

In line with these results, a more recent meta-analysis of 15 prospective cohort studies that included a large number of subjects (*n* = 1,409,014) from the general population with low CVD risk, showed a reduced risk of mortality from CVD and CHD with increasing intake of dietary fibre [138]. Overall, participants who had a high intake of dietary fibre (mean 29.6 g/day for CVD mortality and 23.2 g/day for CHD mortality) had a reduction in CVD mortality by 23% and CHD mortality by 24%, respectively, compared with those who had a low intake of dietary fibre (mean 14.0 g/day for CVD mortality and 12.5 g/day for CHD mortality). This dose-response meta-analysis also showed that a 10 g/day increase of dietary fibre intake was inversely associated with a reduction in mortality from CVD by 9% and from CHD by 11%. Consistent with previous studies, results from this meta-analysis also reported that cereal fibre had a stronger inverse association with risks of CVD and CHD than other types of fibre.

Although extensive evidence supports the protective relationship of dietary fibre, especially fibre from cereals with CVD and CHD risk, it is difficult to determine causality of this association because of the observational nature of the studies on this topic and the lack of long-term randomized controlled trials on patients with CVD.

### 7.1. Possible Mechanisms of Fibre Effects on Cardiovascular Disease 

There are many potential mechanisms through which dietary fibre may act on individual cardiovascular risk factors, i.e., hypertension, central obesity, insulin resistance, and dyslipidemia, which have been discussed in detail in the above paragraphs. Moreover, in addition to fibre, many other potentially beneficial compounds within high fibre foods (fruit, vegetables, whole-grain cereals) could have protective effects (such as antioxidants, phytosterols, vitamins and microelements), so that the combination of these bioactive compounds could have a synergistic beneficial action against CVD.

### 7.2. Conclusion

The overall body of evidence from epidemiological studies suggests that a greater dietary fibre intake, especially cereal fibre, is associated with a lower risk of both CVD and CHD, and death. These findings support the current dietary recommendations to increase the intake of dietary fibre, through an increase of plant-based foods as part of a healthy diet.

## 8. Conclusions

In conclusion, although not conclusively, evidences globally show that dietary fibre given in particular in the context of a fibre-enriched diet may beneficially influence all parameters of body weight management and metabolic disorders related to obesity with consequent advantages on global cardiovascular risk.

Evidence strength varies among metabolic conditions with some inconsistences between observational studies and clinical trials due to differences in duration of trials, selected populations, and type and amount of fibre consumed. Most of high-fermentable (inulin, β-glucan, glucomannan, guar gum and pectin) and some other low-fermentable fibres (psyllium and hydroxypropylmethylcellulose, HPMC) have been shown to significantly reduce plasma LDL cholesterol levels and improve blood pressure control (β-glucan and psyllium), while only high-fermentable fibres showed a beneficial effect on body weight and insulin resistance.

Moreover, low-fermentable fibre has also been shown to improve blood glucose and postprandial triglyceride levels. Evidence from RCT shows that dietary fibre does not affect HDL-cholesterol and fasting triglyceride plasma levels in clinical trials, while less explored are the effects on non-alcoholic fatty liver disease.

No trials explored the specific effects of fibre on cardiovascular events. However, intervention studies exploring the effects of dietary patterns characterized by a high dietary fibre intake such as the Mediterranean Diet show a reduction in cardiovascular risk in people adhering to this lifestyle approach [139].

**Figure 2:**
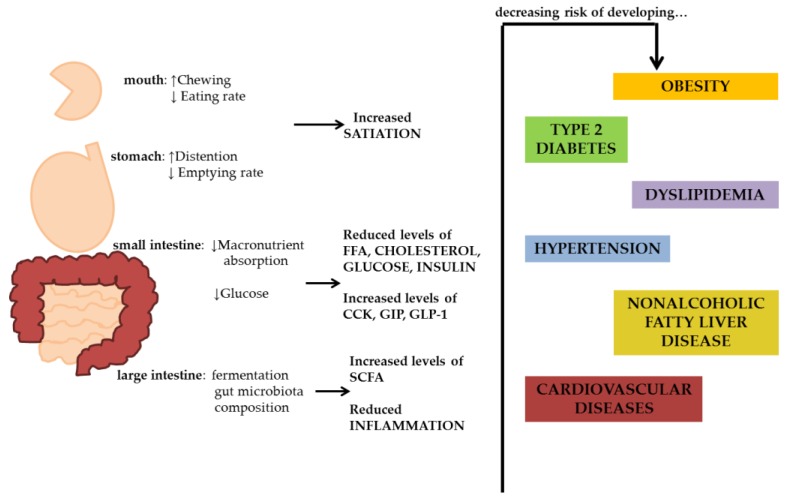
Plausible mechanisms of action whereby fibre may influence body weight and its related common chronic diseases.CCK: cholecystokinin; GIP: gastric inhibitory peptide; GLP-1: glucagon like peptide 1; SCFA: short chain fatty acids.

Dietary fibres act through different pathophysiological mechanisms involving different sites of action (Figure 2). In particular, dietary fibre increases satiety by different effects at the level of the gastrointestinal tract, contributing to disrupt mechanisms leading to positive energy balance. Another relevant pathway is the production of SCFA, that improving both adipose tissue and hepatic insulin resistance acts on a key metabolic crossway from which the pathogenesis of all obesity-associated cardiovascular risk starts. Each of these effects differently influences cardio-metabolic health outcomes. However, this plurality of effects and functionalities suggests that a dietary approach including foods containing all types of fibre would be suitable to globally prevent and treat obesity and related disorders.

Although many of the nutritional studies reported above were designed to induce weight loss, beneficial effects of fibre were also observed in isocaloric conditions or in normal-weight individuals. This suggests that fibres may act on metabolic disorders independently of their weight-lowering effects. Therefore, an isocaloric dietary approach in which foods rich in saturated fatty acids and simple sugars are substituted with fibre-rich food is suitable for managing cardiometabolic risk in obese people non-compliant to calorie-restricted dietary regimen.

Moreover, it should be taken into account that rich-fibre foods are also rich in other beneficial components, such as polyphenols, that may have contributed to the beneficial effects observed on individual cardiovascular risk factors.

## Figures and Tables

**Table 1:** Functional properties and main sources of dietary fibres, according to McRorie et al. [15]

Fibres	Functional Properties			Main Sources
	Viscosity	Solubility	Fermentation	
Bran	Low	Low	Low	Wholegrain
Cellulose				Vegetables
Hemicellulose				Vegetables
Lignin				Seeds
Inulin	Low	High	High	Roots and tubers
Dextrin				Chemically altered wheat and corn starch
Oligosaccharides				Fruits, vegetables, legumes, grains
Resistant starch				Type I: Wholegrain; Type II: High-amylose maize starch, raw potato and banana; Type III: Cooked and cooled starchy foods; Type IV: Chemically modified starches; Type V: Amylose-lipid complex
Pectin	High	High	High	Fruits, vegetables, legumes
*β*-Glucan				Oat and barley
Glucomannan				Tuberous roots of the *Konjac* plant
Guar gum				Leguminous seed plants (guar, locust bean), seaweed extracts (carrageenan, alginates), microbial gums (xanthan, gellan)
*Psyllium*	High	High	Low	Husks of ripe seeds from *Plantago ovate*
Methylcellulose				Food additive

**Table 2:** Published meta-analyses of RCTs evaluating the effects of different types of fibre on body weight regulation.

Author, Year [Reference]	Study Design	Study Population Participants Age BMI Health Status	Intervention and Doses	Duration Weeks	Observed Effects
Pol, 2013 [28]	Meta-analysis of 26 RCTs	2060 M/F 18–70 years 18.5–35.8 kg/m^2^ Healthy	Whole grain (mean dose: 84.1 g/day) vs. refined grain	2–16	= BW ↓ body fat: −0.48% vs. refined grain
Kim, 2016 [29]	Meta-analysis of 21 RCTs	940 M/F 47–57 years 27.6–31.4 kg/m^2^ Metabolic syndrome	Dietary pulses (mean dose: 142 g/day) vs. control	4–12	↓ BW: −0.34 kg vs. control
Mytton, 2014 [30]	Meta-analysis of 8 RCTs	1026 M/F 30–75 years 23.7–37.8 kg/m^2^ Healthy	High fruit and vegetables vs. low fruit and vegetables consumption (mean dose difference in vegetables and fruit consumption between arms 133 g/day)	4–52	↓ BW: −0.54 kg vs. low fruit and vegetables
Thompson, 2017 [31]	Meta-analysis of 12 RCTs	609 M/F 20–70 years 25–35 kg/m^2^ Healthy	Soluble fibre supplementation (mean dose: 18.5 g/day) vs. control	2–17	↓ BMI: −0.84 kg ↓ BW: −2.52 kg ↓ body fat: −0.41% vs. control

= No significant difference; ↓ significant decrease; BMI: body mass index; BW: body weight; F: female; M: male; n.a.: not available; RCT: randomized, controlled trial; WC: waist circumference.

**Table 3:** Published RCTs evaluating the effects of fibre from whole grain or other sources on insulin resistance.

Author, Year [Reference]	Study Design	Study Population Participants Age BMI Health Status	Intervention and Doses	Duration Weeks	Observed Effects
**Fibre from whole grain**					
Pereira, 2002 [46]	RCT	11 M/F 41.6 years 30.2 kg/m^2^ Hyperinsulinemic	Whole-grain products (fibre 28 g/day) vs. refined-grain products (fibre 18 g/day)	6	↑ Insulin sensitivity (euglycemic hyperinsulinemic clamp tests): +0.07 × 10^−4^ mmol·kg^−1^·min^−1^ per pmol/L = BW vs. refined-grain products
Juntunen, 2003 [47]	RCT	20 F 59 years 28 kg/m^2^ Healthy	Rye whole-grain bread (fibre 46 g/day) vs. White wheat bread (fibre 14 g/day)	8	= Insulin sensitivity (FSIGT) = BW vs. White wheat bread
McIntosh, 2003 [48]	RCT	28 M 40–65 years 30 kg/m^2^ Healthy	Rye whole-grain diet (fibre 32 g/day) vs. Wheat whole-grain diet (fibre 32 g/day) vs. Low fibre diet (fibre 19 g/day)	4	= Insulin resistance (HOMA) = BW vs. Low fibre diet
Andersson, 2007 [49]	RCT	30 M/F 59 years 28.3 kg/m^2^ Healthy	Whole-grain products (fibre 18 g/day) vs. refined-grain products (fibre 6 g/day)	6	= Insulin sensitivity (euglycemic hyperinsulinemic clamp tests) ↑ BW vs. refined-grain products
Katcher, 2008 [50]	RCT	47 M/F 46 years 36 kg/m^2^ Metabolic syndrome	Whole-grain products (fibre 12.9 g/1000 kcal) vs. refined-grain products (fibre 9.7 g/1000 kcal)	12	= Insulin sensitivity (ISI index during OGTT) ↓BW vs. refined-grain products
Giacco, 2010 [51]	RCT	15 M/F 55 years 27 kg/m^2^ Healthy	Whole-grain products (fibre 32 g/day) vs. refined-grain products (fibre 20 g/day)	3	= Insulin resistance (HOMA) = BW vs. refined-grain products
Brownlee, 2010 [52]	RCT	216 M/F 46 years 30 kg/m^2^ Healthy	Whole-grain products (60 g/day) vs. Whole-grain products (120 g/day) vs. refined-grain products	16	= Insulin sensitivity (QUICKI) = BW vs. refined-grain products
Giacco, 2013 [53]	RCT	133 M/F 40–65 years 31.4 kg/m^2^ Metabolic syndrome	Whole-grain products (fibre 33 g/day) vs. refined-grain products (fibre 20 g/day)	12	= Insulin sensitivity (FSIGT) = BW vs. refined-grain products
Giacco, 2014 [54]	RCT	54 M/F 40–65 years 31.7 kg/m^2^ Metabolic syndrome	Whole-grain products (fibre 33 g/day) vs. refined-grain products (fibre 20 g/day)	12	= Insulin resistance (HOMA) = BW vs. refined-grain products
**Fibre from other sources**					
He, 2016 [55]	Meta-analysis of 18 RCTs	298 M/F 53 years 26 kg/m^2^ Any	Oat-based products (20–136 g/day) vs. β-glucan extract (3–10 g/day) vs. refined-grain products	4-12	= Insulin resistance (HOMA) = BW vs. refined-grain products
Hashizume, 2012 [56]	RCT	30 M/F 60.6 years 72.5 kg/m^2^ Metabolic syndrome	Resistant maltodextrin (27 g/day) vs. placebo	12	↓ Insulin resistance (HOMA): −0.5% = BW vs. placebo
Li, 2010 [57]	RCT	120M 31 years 24.5 kg/m^2^ Healthy	NUTRIOSE * (27 g/day) vs. placebo	12	↓ Insulin resistance (HOMA): −12% ↓ BW: −1.5 kg
Johnston, 2010 [58]	RCT	20 M/F 47.6 years 30.8 kg/m^2^ Metabolic syndrome	Resistant starch (40 g/day) vs. placebo	12	↑ Insulin sensitivity (euglycemic hyperinsulinemic clamp tests tests): +0.9 mg·kg^−1^·min^−1^ per pmol/L = BW vs. placebo
Robertson, 2012 [59]	RCT	15 M/F 48.9 years 33.8 kg/m^2^ Metabolic syndrome	Resistant starch (40 g/day) vs. placebo	8	↓ Insulin resistance (HOMA): −0.4% = BW vs. placebo

= No significant difference; ↓ significant decrease; ↑ significant increase; BMI: body mass index; BW: body weight; HOMA: Homeostatic model assessment; ISI: insulin sensitivity index; F: female; FSIGT: Frequently sampled intravenous glucose tolerance test; M: male; n.a.: not available; NSP: nonstarchy polysaccharides; OGTT: oral glucose tolerance test; QUICKI: Quantitative insulin sensitivity check index. * NUTRIOSE is a soluble fibre.

**Table 4:** Published meta-analyses of RCTs evaluating the effects of different types of fibre on blood glucose control.

Author, Year [Reference]	Study Design	Study Population Participants Age BMI Health Status	Intervention and Doses	Duration Weeks	Observed Effects
Post, 2002 [79]	Meta-analysis of 15 RCTs	400 M/F 52–69 years 23.4–32.5 kg/m^2^ Type 2 diabetes	High fibre diet vs. low-fibre diet (4–40 g/day)	3–12	↓ Fasting glucose:−15 mg/dL ↓ HbA1c: −0.26% vs. low-fibre diet (4–40 g/day)
Silva, 2013 [80]	Meta-analysis of 13 RCTs	605 M/F 62 years n.a. Type 2 diabetes	High fibre foods vs. low-fibre foods (3–22.5 g/day)	8–24	↓ Fasting glucose:−9.97 mg/dL ↓ HbA1c: −0.55% vs. low-fibre foods
Gibb, 2015 [81]	Meta-analysis of 35 RCTs	1075 M/F 52.3 years n.a. Type 2 diabetes	*Psyllium* (3–10 g/day) vs. placebo	2–26	↓ Fasting glucose: −37 mg/dL ↓ HbA1c: −0.97% vs. placebo
Schwingshackl, 2018 [82]	Meta-analysis of 56 RCTs	4937 M/F 44–67 years 25–43 kg/m^2^ Type 2 diabetes	Mediterranean diet vs. control diet	3–48	↓ Fasting glucose: −11.0 mg/dL ↓ HbA1c: −0.32% vs. control diet
Marventano, 2017 [83]	Meta-analysis of 14 RCTs	377 M/F 50 years 28 kg/m^2^ Healthy	Whole-grain products vs. refined-grain products	2–16	= Fasting glucose = Fasting insulin vs. refined-grain products
Thompson, 2017 [31]	Meta-analysis of 12 RCTs	200 M/F 20–70 years 25–45 kg/m^2^ Healthy	Soluble fibre supplementation (3–34 g/day) vs. Non-fibre placebo	2–17	↓ Fasting glucose: −3.0 mg/dL ↓ Fasting insulin: −2.29 μU/mL ↓ BW: −2.52 kg vs. Non-fibre placebo

= No significant difference; ↓ significant decrease; ↑ significant increase; BMI: body mass index; BW: body weight; F: female; M: male.

**Table 5:** Published meta-analyses of RCTs evaluating the effects of fibre from whole grain or other sources on serum lipids and two clinical trials also evaluating the postprandial lipid response.

Author, Year [Reference]	Study Design	Study Population Participants Age BMI Health Status	Intervention and Doses	Duration Weeks	Observed Effects
**Fibre from whole grain**					
Ye, 2012 [89]	Meta-analysis of 21 RCTs	1281 M/F 20–74 years n.a. Healthy/Hypertension	Whole-grain diet vs. control diet	4–16	↓ TC: −32 mg/dL ↓ LDL-C: −28 mg/dL = BW vs. control
Hollander, 2015 [90]	Meta-analysis of 24 RCTs	2275 M/F 18–75 years ≥18 kg/m^2^ Dyslipidemia	Whole-grain products (fibre 20 g/day) vs. refined-grain products (fibre 14 g/day)	2–16	↓ TC: −5.0 mg/dL ↓ LDL-C: −3.0 mg/dL No effect on HDL-C and TG = BW vs. refined products
Kelly, 2017 [92]	Meta-analysis of 9 RCTs	1414 M/F 24–70 years n.a. Any	Whole-grain products (fibre 21 g/day) vs. control products (fibre 13 g/day)	12–16	No effect on TC and LDL-C = BW vs. control products
Giacco, 2014 [54]	RCT	54 M/F 57 ± 8 years 32 ± 5kg/m^2^ Metabolic syndrome	Whole-grain products (fibre 40 g/day) vs. refined-grain products (fibre 22 g/day)	12	Postprandial TG (IAUC) −43% = BW vs. refined products
**Fibre from other sources**					
Hartley, 2016 [91]	Meta-analysis of 23 RCTs	1513 M/F ≥18 years n.a. High CV risk	Fibre supplementation (1.2–27.5 g/day) vs. control diet	12	↓ TC: −8.0 mg/dL ↓ LDL-C: −5.0 mg/dL No effect on TG = BW vs. control
Whitehead, 2014 [93]	Meta-analysis of 28 RCTs	1914 M/F 25–63 years Any	Oat β-glucan supplementation (≥3 g/day) vs. refined products	2–12	↓ TC: −12 mg/dL ↓ LDL-C: −10 mg/dL vs. refined products
Zhu, 2015 [94]	Meta-analysis of 17 RCTs	916 M/F 54 years 27 kg/m^2^ Hypercholesterolemia	Oat/Barley β-glucan-rich diet (5 g/day) vs. control diet	7	↓ TC: −10 mg/dL ↓ LDL-C: −8.0 mg/dL No effect on HDL-C and TG vs. control
Ho, 2016 [95]	Meta-analysis of 14 RCTs	723 M/F 47 years 26.1 kg/m^2^ Any	Barley β-glucan-rich diet (6 g/day) vs. control diet	4	↓ LDL-C: −10 mg/dL vs. control
Ho, 2016 [96]	Meta-analysis of 56 RCTs	3745 50 years 27 kg/m^2^ Any	Oat β-glucan-rich diet (4 g/day) vs. control diet	6	↓ LDL-C: −7.0 mg/dL vs. control
Bazzano, 2011 [97]	Meta-analysis of 10 RCTs	268 M/F 18–66 years n.a. Dyslipidemia	Non-Soy Legume diet (80–440 g/day; fibre 23 g/day) vs. control diet (fibre 18 g/day)	3–8	↓ TC: −11.8 mg/dL ↓ LDL-C: −8.0 mg/dL No effect on HDL-C and TG = BW vs. control
Ha, 2014 [99]	Meta-analysis of 26 RCTs	1037 M/F 29–64 years ≥18 kg/m^2^ Any	Legume-rich diet (130 g/day; fibre 26 g/day) vs. control diet (fibre 20 g/day)	3–48	↓ LDL-C: −7.0 mg/dL No effect on HDL-C = BW vs. control
Liu, 2017 [101]	Meta-analysis of 20 RCTs	607 M/F 18–67years 19–39 kg/m^2^ Any	Inulin-type fructans supplementation (7.4–30 g/day) vs. control diet	2.5–24	↓ LDL-C: −6.0 mg/dL = BW vs. control
De Natale, 2009 [102]	RCT	18 M/F 59 ± 5 years 27 ± 3 kg/m^2^ Type 2 diabetes	High-Carbohydrate/High-Fibre diet (fibre 28 g/1000 kcal) vs. High–MUFA/Low-Carbohydrate diet (fibre 8 g/1000 kcal)	4	Postprandial TG (IAUC) −31% = BW vs. High–MUFA/Low-Carbohydrate diet

= No significant difference; ↓ significant decrease; BMI: body mass index; BW: body weight; F: female; HDL-C: High-density lipoprotein cholesterol; IAUC: Incremental area under the curve; LDL-C: Low-density lipoprotein cholesterol; M: male; MUFA: monounsaturated fatty acids; n.a.: not available; TC: total cholesterol; TG: Triglycerides.

**Table 6:** Published meta-analyses of RCTs evaluating the effects of different types of fibre on blood pressure.

Author, Year [Reference]	Study Design	Study Population Participants Age BMI Health Status	Intervention and Doses	Duration Weeks	Observed Effects
Streppel, 2005 [115]	Meta-analysis of 24 RCTs	1404 M/F 23–63 years n.a. normotensive and hypertensive	Soluble/insoluble fibre supplementation (mean dose: 11.5 g/day) vs. placebo	2–24	↓ SBP: −1.13 mm Hg ↓ DBP: −1.26 mmHg = BW vs. placebo
Hartley, 2016 [91]	Meta-analysis of 23 RCTs	1513 M/F ≥18 years n.a. any	Soluble/insoluble fibre supplementation (1.2–27.5 g/day) vs. control diet	12	↓ DBP: −1.77 mmHg = BW vs. control diet
Whelton, 2005 [116]	Meta-analysis of 25 RCTs	1477 M/F 16–85 years n.a. normotensive and hypertensive	Fruit/vegetables/cereals/pectins/ guar gum rich diets or supplementation (10.7 g/day) * vs. control diet/placebo	2–26	↓ DBP: −1.65 mm Hg In hypertensive subjects: ↓ SBP: −5.95 mm Hg ↓ DBP: −4.20 mmHg ↓ BW in n.9 RCTs vs. control diet/placebo
Evans, 2015 [117]	Meta-analysis of 28 RCTs	1333 M/F 29–60 years n.a. healthy	Whole oats, oat bran-supplemented foods or oat-based breakfast cereals (mean dose: 4 g/day) vs. wheat-based foods	6–14	↓ SBP: −2.7 mm Hg ↓ DBP: −1.5 mmHg = BW vs. wheat-based foods
Khan, 2018 [118]	Meta-analysis of 22 RCTs	1430 M/F 15–69 years n.a. any	Viscous fibre supplementation (b-glucan from oats and barley, guar gum, konjac, pectin and psyllium supplementation) (fibre 8.7 g/day) vs. control products	4–24	↓ SBP: −1.59 mm Hg ↓ DBP: −0.39 mmHg *Psyllium* fibre supplementation: ↓ SBP: −2.39 mm Hg = BW vs. control products

* Doses are reported as mean difference in intake between intervention vs. control groups; = no significant difference; ↓ significant decrease; BMI: body mass index; BW: body weight; DBP: diastolic blood pressure; F: female M: male; n.a.: not available; SBP: systolic blood pressure.
